# Antiallodynic effects of KDS2010, a novel MAO-B inhibitor, via ROS-GABA inhibitory transmission in a paclitaxel-induced tactile hypersensitivity model

**DOI:** 10.1186/s13041-022-00924-9

**Published:** 2022-05-07

**Authors:** Su Eun Park, Chiranjivi Neupane, Chan Noh, Ramesh Sharma, Hyun Jin Shin, Thuy Linh Pham, Gyu-Seung Lee, Ki Duk Park, C. Justin Lee, Dong-Wook Kang, So Yeong Lee, Hyun-Woo Kim, Jin Bong Park

**Affiliations:** 1 Department of Medical Sciences, Graduate School, Chungnam National University, Daejeon, 35015 Korea; 2 Department of Physiology, College of Medicine and Brain Research Institute, Chungnam National University, Daejeon, 35015 Korea; 3 Department of Anesthesiology and Pain Medicine, Chungnam National University Hospital, Daejeon, 35015 South Korea; 4 Dong-Gu Health Promotion Center 301-01, 30 Bogeunso Avenue, Samseung-Dong, Dong-gu, Daejeon, South Korea; 5 Brain Science Institute, Korea Institute of Science and Technology (KIST), Seoul, 02792 Korea; 6 Center for Cognition and Sociality, Institute for Basic Science, Daejeon, 34126 Korea; 7 Laboratory of Veterinary Pharmacology, College of Veterinary Medicine and Research Institute for Veterinary Science, Seoul National University, Seoul, 08826 Korea

**Keywords:** KDS2010, MAO-B, Tactile Hypersensitivity, Paclitaxel

## Abstract

Monoamine oxidase (MAO) inhibitors have been investigated for the treatment of neuropathic pain. Here, we assessed the antiallodynic effects of a novel MAO-B inhibitor, KDS2010, on paclitaxel (PTX)-induced mechanical hypersensitivity. Oral administration of KDS2010 effectively relieved PTX-induced mechanical hypersensitivity in a dose-dependent manner. KDS2010 (25 mg/Kg) significantly prevented and suppressed PTX-induced pain responses with minimal effects on the body weight, motor activity, and working memory. KDS2010 significantly reduced reactive astrocytosis and reactive oxygen species (ROS) level in the L4–L6 spinal cord of PTX-treated mice. Furthermore, KDS2010 reversed the attenuation of GABAergic spontaneous inhibitory postsynaptic current (sIPSC) frequency in spinal dorsal horn neurons, although it failed to restore the reduced tonic GABA_A_ inhibition nor the increased GABA transporter 1 (GAT1) expression in PTX-treated mice. In addition, bath application of a reactive oxygen species (ROS) scavenger (PBN) restored the sIPSC frequency in PTX-treated mice but not in control and PTX + KDS2010-treated mice. These results indicated that the antiallodynic effect of KDS2010 is not due to a MAO-B-dependent GABA production. Finally, PBN alone also exerted a similar analgesic effect as KDS2010, but a co-treatment of PBN with KDS2010 showed no additive effect, suggesting that inhibition of MAO-B-dependent ROS production is responsible for the analgesic effect by KDS2010 on PTX-induced allodynia. Overall, KDS2010 attenuated PTX-induced pain behaviors by restoring the altered ROS level and GABAergic inhibitory signaling in the spinal cord, suggesting that KDS2010 is a promising therapeutic strategy for chemotherapy-induced peripheral neuropathy.

## Introduction

Chemotherapy-induced peripheral neuropathy (CIPN), including mechanical hypersensitivity, is a common dose-limiting side effect of many chemotherapeutic agents [1, 2]. Paclitaxel (PTX), one of the most effective chemotherapeutic drugs, is widely used for the treatment of solid tumors, including in ovarian, breast, and non-small-cell lung cancer [3]. The use of PTX is often associated with neurotoxicity, which typically results in sensory dysfunction and pain [4, 5]. Due to the limited effective treatments, a better understanding of the mechanism, and the discovery of new and effective strategies to treat mechanical allodynia, are matters of great urgency.

Monoamine oxidases (MAOs), which have two isoforms, A and B, are flavor enzymes located in the outer membrane of mitochondria that metabolize monoaminergic neurotransmitters in the central nervous system (CNS). During the process of oxidative deamination, reactive oxygen species (ROS) are constantly generated as potential deleterious by-products. Oxidative stress, i.e., the imbalance between ROS production and detoxification of its harmful effects, has been implicated in the development of various conditions characterized by persistent pain [6–10]. MAO inhibitors have been explored for pain treatment since their initial use for the treatment of pain associated with angina pectoris. Thus, MAO inhibitors may represent a treatment alternative for neuropathic pain [11–13], although the mechanism remains unclear.

KDS2010, a newly developed reversible MAO-B inhibitor [14], restores the altered GABAergic inhibition seen in brain diseases [14, 15]. ROS involvement in neuropathic pain is related to diminished inhibitory GABAergic transmission of substantia gelatinosa (SG) neurons [10, 16]. Altered tonic GABA_A_ inhibition also contributes to PTX-induced CIPN [17, 18]. These observations prompted us to assess whether the MAO-B inhibitor improves neuropathic pain and reverses the altered GABAergic inhibition. Here, we demonstrated that KDS2010 relieved PTX-induced mechanical hypersensitivity in a ROS-dependent manner, along with restoration of attenuated GABA_A_ synaptic transmission, indicating that MAO-B inhibition has therapeutic potential for CIPN.

## Materials and methods

### Animals

Male ICR mice (6 weeks old), purchased from Samtako Bio (Korea) were used for experiments. All animals were maintained on 12 h alternating light/dark schedule in a temperature-controlled environment and had free access to normal drinking water and food pellets. All experiments were conducted in accordance with the ethical guidelines of the International Association for the Study of Pain and were approved by the Animal Care and Use Committee at the Chungnam National University (202006A-CNU-086).

### Experiment procedure

Animals were divided into three groups (i) control group (Con) for animals with 5% DMSO injection I.P for 5 successive days, (ii) Paclitaxel group (PTX) for animals with PTX injection,, and (iii) the KDS2010 group (PTX + KDS) for animals with KDS2010 treatment. Paclitaxel (PTX, Sigma, St. Louis, MO, USA) dissolved in 5% dimethyl sulfoxide (DMSO, Sigma, St. Louis, MO, USA) was treated at a dose of 4 mg/kg (i.p.) once a day for 5 consecutive days. KDS2010 dissolved in distilled water was treated per orally (7.5 mg/kg-50 mg/kg, P.O., BID) for 14 consecutive days (unless otherwise mentioned) before or after the induction of tactile hypersensitivity with PTX. Phenyl-N-*t*-butylnitrone (PBN, Sigma, St. Louis, MO) dissolved in physiological saline solution was treated at a dose of 100 mg/kg (i.p).

### Behavior assessment

#### Mechanical hypersensitivity test

The number of paw withdrawal responses to normally innocuous mechanical stimuli was measured by using a von Frey filament (2.0 g, North Coast Medical, Morgan Hill, CA, USA) 2 day prior to drugs treatment and in every 2 days after drugs injection as described in a previous study [19]. Briefly, mice were placed on a metal mesh grid under a chamber, and the von Frey filament was applied from underneath the metal mesh flooring to each hind paw. They were then left alone for at least 30 min of acclimation before sensory testing began. The von Frey filament was applied 10 times to each hind paw, and the number of paw withdrawal responses out of 10 was expressed as percent withdrawal responses out of 10.

#### Rotarod test

Mice were placed on a long cylindrical rod, which rotates along its long axis during a 5 min session. The speed of the rod held constant in 4 rpm. When mice fall off from the rod onto the plate placed below, the animal latency to fall (in seconds) was recorded. The length of time the animal stays on this rotating rod is a measure of their balance, coordination, physical condition, and motor planning.

#### Open field test

 The open field consists of a square acrylic box (40 × 40 cm). The open field sessions were videotaped from the top of the arenas in a dimly illuminated room. To monitor locomotor activity in a novel whole field (40 × 40 cm) and a novel central field (20 × 20 cm), mice were placed in a novel open field for 30 min and the activity was analyzed using Ethovision® XT (Noldus, Wageningen, The Netherlands).

#### Y-maze test

The maze consists of three arms (34 cm long, 6 cm wide and 14.5 cm deep, labeled A, B, or C) diverging at a 120° angle from the central point. The experiments were performed in a dimly illuminated room, and the floor of the maze was cleaned with 70% ethanol-soaked paper after each mouse was tested. Each mouse was placed at center and allowed to move freely in the maze during an 8-min session. The sequence of arm entries was counted manually. The spontaneous alternation consists of sequential entries into all three arms (e.g., the sequence, ABCBCBCA was counted two with the first sequential ABC and the last sequential BCA).Then, the percentage of spontaneous alteration was calculated by dividing the number of alteration by the number of possible alterations [number of alternations/ (number of total arm entries-2)].

#### Detection of ROS

To detect the generation of ROS in the spinal cord, dihydroethidium (DHE) staining was performed as previously described [20]. Animals were deeply anesthetized with avertin (250 mg/kg, i.p; Sigma Aldrich, St. Louis, MO) and perfused with heparinized phosphate buffered saline (PBS, pH 7.4) on day 12^th^ of KDS2010 and PTX co-treatment. The perfusion was followed by 4% paraformaldehyde in PBS for 10 min using a peristaltic pump at a rate of 20 ml/min. The lumbar spinal cord (L4-L6) was collected and immersed in the same fixative for 2 h. The tissues were then cryoprotected in a series of sucrose solutions (10–30%) for 2 days. Next, spinal cords were embedded in Tissue-Tek O.C.T. compound and then frozen immediately in liquid nitrogen. Spinal cords were cut into 30 μm thick transverse sections and were then incubated with 1 μM DHE (Thermo Fisher Scientific) at room temperature for 5 min and mounted on slides. The red fluorescence was detected using a fluorescence microscope (Axio Scope A1; Zeiss, Germany) and recorded with a digital camera (AxioCam MRm; Zeiss).

For quantitative analysis of red fluorescence, three spinal cord sections from each animal group were randomly selected and quantified in the dorsal horn regions using Image J software (NIH, Bethesda, MD, USA).

### Immunofluorescence

The lumbar region (L4–L6) of the spinal cord was isolated from the animals anesthetized and perfused with heparinized PBS and followed by 4% paraformaldehyde in PBS. Spinal cord was kept overnight for post fixation in same fixative at 4℃, followed by immersion in a series of sucrose solutions in PBS (10% to 30%) for cry protection [21]. Next, spinal cord were embedded in O.C.T compound and stored at -70℃. After 2 days, frozen Sects. (30 µM) encompassing lumbar spinal cord were prepared using a cryostat (CM1950; Leica Microsystems, Wetzlar, Germany) and kept at 4 °C in a storage buffer (30% glycerol, 30% ethylene glycol in PBS).

For immunofluorescence staining, tissues were blocked with a blocking solution (3% BSA, 0.1% Triton X-100 in 1X PBS) for 1 h at room temperature. Afterwards, the tissues were incubated with an of primary antibodies against GFAP (Abcam, Cat. No. ab53554, 1:200) diluted in the blocking buffer overnight at 4℃. Next day, sections were treated with FITC conjugated secondary antibody (Abcam, Cat. No. ab97050, 1:200) diluted in the same blocking buffer [22]. Finally, the sections were counterstained and mounted with DAPI (Thermo Fisher Scientific, Waltham, MA, USA) on glass slides. Images were visualized with microscopy (Axio Scope A1; Zeiss, Germany) and images captured by using a digital camera (AxioCam MRm; Zeiss).

### Western blot analysis

The spinal cord was extracted by pressure expulsion with air into an ice-cooled, PBS (Phosphate buffered saline)-filled glass dish and snap frozen in liquid nitrogen. Lumber 4–6 segments of the spinal cord were homogenized with RIPA buffer (20 mM Tris-HCI, pH 7.5, 150 mM NaCl, 1 mM EDTA, 1 mM EGTA, 1% NP-40, 1% sodium deoxycholate, 2.5 mM sodium pyrophosphate, 1 mM β-glycerophosphate, 1 mM Na_3_VO_4_, 1 µg/ml 1eupeptin) containing protease inhibitor, 0.1% SDS (Sodium dodecyl sulfate) and the insoluble materials were removed by centrifugation at 12,000 rpm for 10 min at 4 °C. Protein concentrations were determined using BCA assay. Lysates of the spinal cord were separated by 10% SDS-PAGE (Sodium dodecyl sulfate–polyacrylamide) gel for electrophoresis and then transferred to a nitrocellulose membrane. Nonspecific binding was blocked with 3% Bovine Serum Albumin (Sigma, St. Louis, MO, USA) in TBST (0.1% Tween 20 in 1X TBS) for 1 h at room temperature. The membrane was washed with TBST three times, for 10 min each time, and incubated with primary antibodies against the GFAP (goat, IgG, Abcam, Cat. No. ab53554, 1:5000), GAT1 (rabbit, IgG, Abcam, Cat. No. ab426, 1:10,000 and β-actin (mouse, IgG, sigma, Cat. No. A5441, 1:5000) for overnight at 4 °C. Next, the membranes were incubated with secondary antibody diluted in the same blocking buffer at room temperature for 2 h. After washing three times with TBST, proteins were detected using an X-ray film imaging system with an enhanced chemiluminescence detection kit (ECL; Pierce, USA). The relative expression levels of protein to β-actin was analyzed using Image J software (NIH, Bethesda, MD, USA).

### Spinal cord slice preparation and electrophysiological recording

Spinal cord slices were obtained from mice as previously described [23]. Briefly, mice were anesthetized with avertin, spinal cord was quickly removed from the vertebral canal by hydraulic extrusion and placed in ice-cold artificial cerebrospinal fluid (aCSF), containing 126 mM NaCl, 5 mM KCl, 1.2 mM MgCl_2_, 26 mM NaHCO_3_, 1.2 mM NaH_2_PO_4_, 10 mM glucose, 2.4 mM CaCl_2_; pH 7.3—7.4 and saturated with 95% O_2_ and 5% CO_2._ Transverse spinal cord slices (300 μm thick) were cut in the ice-cold aCSF and then incubated for 1 h in aCSF at 32 °C before they were transferred to the recording chamber. For patch clamp recording, patch pipettes were filled with internal solution containing (in mM): Cesium methanesulfonate 135, KCl 10, HEPES 10, Mg^2+^ATP 5, MgCl_2_ 0.9, and EGTA 10. In the whole cell configuration, GABAergic current were recorded (at 0 mV) from spinal substancia gelatinosa (SG) neurons in the presence of 6-cyano-7-nitroquinoxaline-2,3-dione disodium (CNQX;10 μM, Tocris), DL-2-Amino-5-phosphonopentanoic acid (DL-AP5; 50 μM, Tocris) and 0.5 μM Strychnine (Sigma) to block glutamate and glycine receptors. Recording were obtained using Multiclamp 700B (Molecular devices). Data were filtered at 1 kHz and digitized at 10 kHz (Digidata 1440A) and acquired using pClamp 11 software. The series resistance was monitored at the beginning and end of the experiments and data were excluded if changes > 20% were observed. The spontaneous inhibitory postsynaptic currents (sIPSCs) were detected and analyzed using Mini analysis (Synaptosoft, Decatur, CA). The GABA_A_ receptor-mediated tonic current was estimated by *I*_holding_ difference before and after application of GABA_A_ receptor antagonist bicuculline (BIC; 20 μM) as in our previous reports [24].

### Statistical analysis

Statistical analysis was performed using Graph Pad Prism 6 (Graph Pad Software). Numerical data are presented as the mean ± standard error of mean (SEM). The statistical significance was determined between the groups by using paired t-test and analysis of variance (ANOVA) followed by a post-hoc Tukey’s test. A value of p < 0.05 was considered to be statistically significant.

## Results

### KDS2010 reduced PTX-induced mechanical hypersensitivity with minimal side effects

**Fig. 1:**
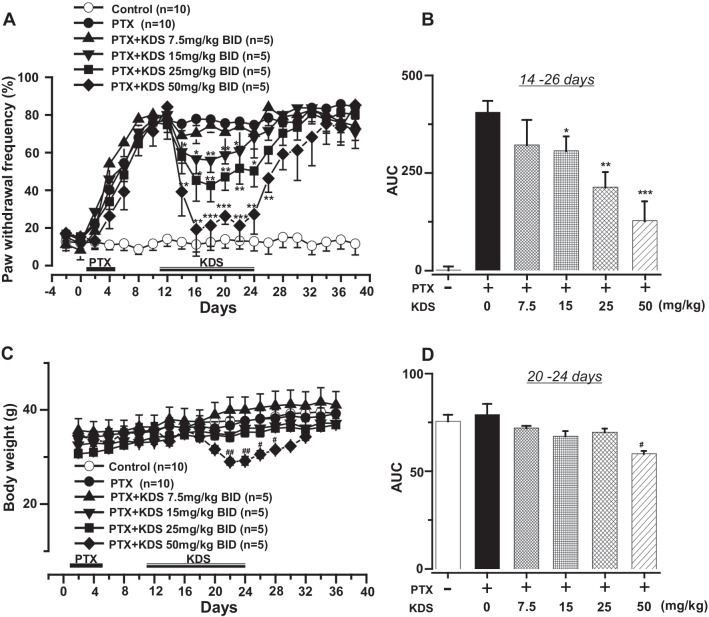
KDS2010 reduced PTX-induced mechanical hypersensitivity with minimal side effects. **A** Paw withdrawal frequency (PWF %) was compared among the control, paclitaxel (PTX), and KDS2010 (PTX + KDS) groups at the indicated time points. PTX induced mechanical hypersensitivity peaked at day 12 after the first injection and continued until at least day 38. KDS2010 significantly reduced PTX-induced mechanical hypersensitivity in a concentration-dependent manner. **B** The area under the curve (AUC) of PWF from 14 to 26 days. **C** The body weight of the mice was monitored throughout the experimental time course. **D** The area under the curve for body weight, from 20 to 24 days. All data are presented as mean ± SEM. *P < 0.05, **P < 0.01, ***P < 0.01 compared to PTX-treated group, #P < 0.05 compared to control

As demonstrated in a previous study of mechanical hypersensitivity [25], PTX (4 mg/kg I.P. once per day for 5 consecutive days) resulted in tactile hypersensitivity, as evidenced by increased paw withdrawal frequency (PWF%) in the von-Frey filament test [25]. PWF ranged from 10 to 20% in the control group, whereas it increased on post-PTX-injection day 4 in PTX-treated mice, reaching approximately 70% on day 6 and remaining at that level until day 40 (Fig. 1A).

We intended to reduce PTX-induced mechanical hypersensitivity via treatment with KDS2010 at the level found to improve cognitive function in Alzheimer disease (25 mg/kg via water intake) [14]. KDS2010 significantly suppressed PTX-induced tactile hypersensitivity at 2 days after treatment, reached its maximal effect at 4 days and maintained that level of effectiveness thereafter throughout the treatment period (data not shown).

Similar antiallodynic effects were observed under P.O. treatment with KDS2010. KDS2010 (7.5–50 mg/kg twice per day) reduced PTX-induced pain responses in a concentration-dependent manner (Fig. 1B). At a concentration of 7.5 mg/kg, KDS2010 did not affect PWF in PTX-treated mice, whereas the higher doses (15, 25, and 50 mg/kg) reduced the PTX-induced pain response. The highest dose of KDS2010 (50 mg/kg) caused significant weight loss (Fig. 1C), which was not the case at the lower concentrations. Thus, 15 or 25 mg/kg KDS2010 was used in subsequent experiments.

### KDS2010 prevented and suppressed PTX-induced tactile hypersensitivity

**Fig. 2:**
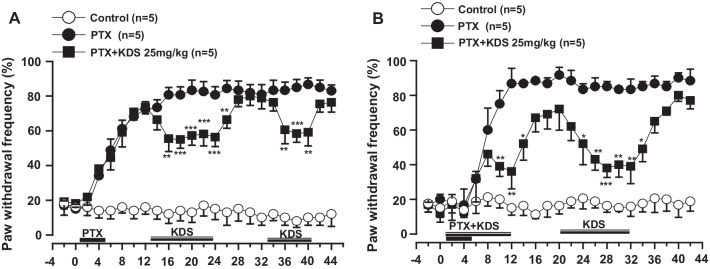
KDS2010 prevented and suppressed PTX-induced tactile hypersensitivity. Frequency of paw withdrawal responses (%) in the von Frey filament tests was measured at different time points in the control, PTX, and KDS2010 (PTX + KDS) groups. **A** KDS2010 had significant reversible effects on PTX-induced tactile hypersensitivity. Similar anti-allodynic effects were observed with the second application of KDS2010 (31–41 days) in the same group. **B** Co-treatment with KDS2010 had similar reversible effects on PTX-induced tactile hypersensitivity, as shown in (**A**). All data are presented as mean ± SEM. *P < 0.05, **P < 0.01, ***P < 0.01 compared to the PTX-treated group

Given that KDS2010 is a reversible MAO-B inhibitor [14], the antiallodynic effects of KDS2010 could be reversible and reproducible. To test this hypothesis, we compared the antiallodynic effects of repeated KDS2010 application, and the withdrawal period, in the same PTX-treated mice (Fig. 2).

As shown in Fig. 1, the antiallodynic effects of KDS2010 were reversible. KDS2010 significantly reduced PTX-induced tactile hypersensitivity, as shown by the significant decrease in PWF from ~ 75% to ~ 50% (Fig. 2A). PWF increased again with withdrawal of KDS2010. The second application of KDS2010 reproduced the maximal antiallodynic effects seen in the same PTX-treated animals (Fig. 2A). The results showed that the minimal PWF did not differ between the first and second applications of KDS2010 (Fig. 2A), suggesting that the antiallodynic effect of KDS2010 was mediated by the reversible inhibition of MAO-B.

Similar results were observed with KDS2010 co-treatment (Fig. 2B). We compared the preventive effects of co-treatment with KDS2010 on PTX-induced tactile hypersensitivity to those on the established PTX-induced pain response (Fig. 2B). Co-treatment with KDS2010 significantly reduced PTX-induced tactile hypersensitivity, as shown by the significant decrease in PWF from ~ 75% in the PTX group to ~ 50% in the KDS2010-pretreated group (P < 0.001, Fig. 2B). PWF increased again after withdrawing KDS2010, and the application of additional KDS2010 efficiently decreased PWF. Thus, PWF did not differ between the co- and post-treated periods (Fig. 2B), suggesting that KDS2010 prevents the development of PTX-induced tactile hypersensitivity, as well as being therapeutically effective for pre-existing PTX-induced pain.

### KDS2010 prevented PTX-induced astrogliosis in the spinal cord

Chronic pain may result from gliopathy [26]. In the next set of experiments, we investigated the effects of KDS2010 on astrogliosis and microgliosis in PTX-induced CIPN mice.

**Fig. 3:**
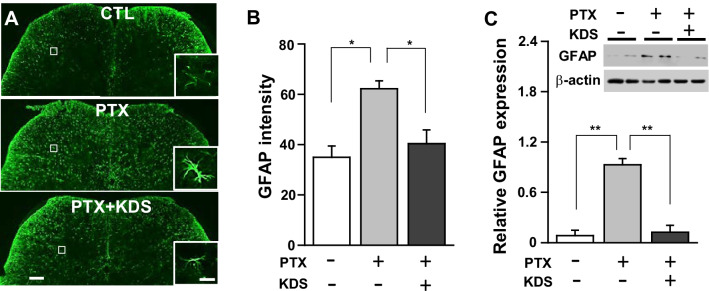
KDS2010 prevented PTX-induced astrogliosis in the spinal cord. **A** Representative Immunostaining images of GFAP in the control, PTX, and KDS2010 (PTX + KDS) groups. Scale bars = 50 µm (left) and 5 µm (right). **B** Summary bar graphs for GFAP intensity. **C** Immunoblots (upper panel) showing GFAP expression in the spinal cord in the control, PTX, and KDS2010 (PTX + KDS) groups, and corresponding quantitative data for relative GFAP expression (lower graph). All data are presented as mean ± SEM. *P < 0.05, **P < 0.01, compared to the control group

We compared astrocytes and microglia among control, PTX-induced CIPN, and PTX co-treated with KDS2010 (PTX + KDS) mouse groups. The microglial activity shown by iba1-ir in the L5 spinal dorsal horn did not differ significantly among the groups (data not shown), indicating that CIPN may not be associated with microgliosis [27, 28]. Meanwhile, astrogliosis was evident in PTX-induced CIPN, as shown by the increased GFAP-ir in the spinal dorsal horn of the PTX groups (Fig. 3A). KDS2010 significantly prevented PTX-induced astrogliosis, as shown by the significant decrease in GFAP-ir in the PTX-KDS2010 compared to PTX group (Fig. 3A, B).

Similar results were seen for GFAP protein expression by Western blot. Increased GFAP protein expression in the spinal cord of PTX-treated mice was significantly attenuated by KDS2010 compared to the control group (Fig. 3C).

### Comparison of the antiallodynic effects of KDS2010 and PBN on tactile hypersensitivity and ROS

**Fig. 4:**
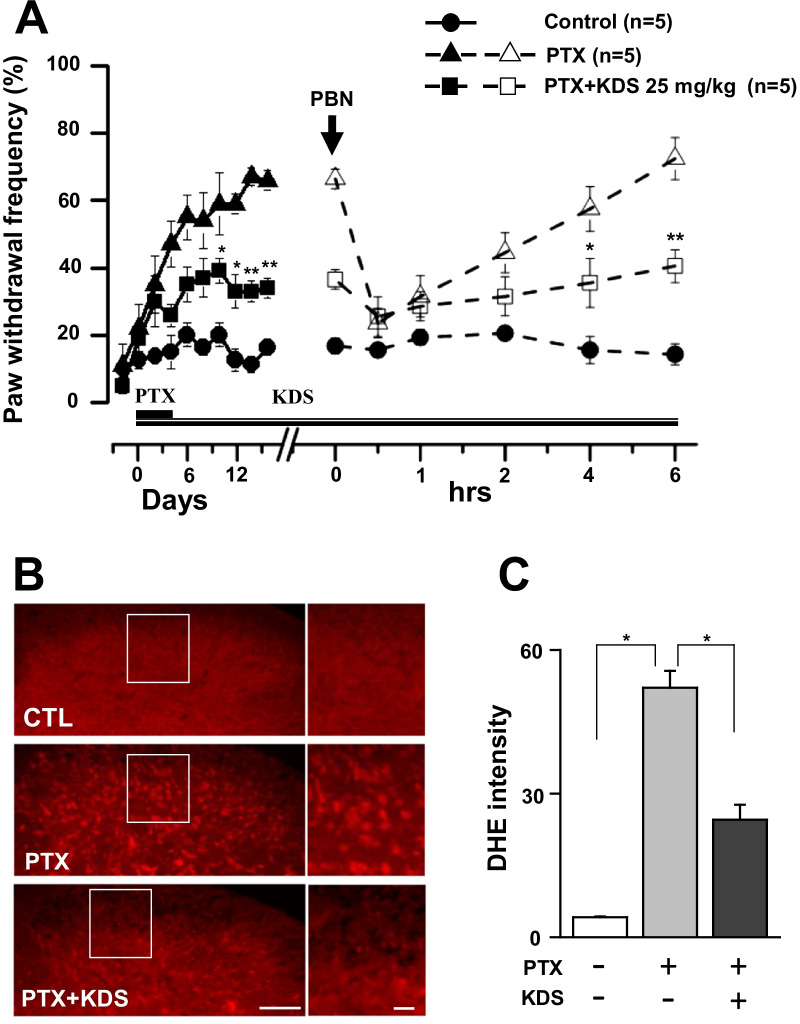
Comparisons of antiallodynic effects of KDS2010 and PBN on tactile hypersensitivity and ROS. **A** Effects of PBN on paw withdrawal (%) responses in PTX- and KDS2010-(PTX + KDS) treated mice. The peak effects of PBN did not differ among the groups. **B** Representative images of DHE staining in the control, PTX-, and KDS2010 (PTX + KDS)-treated mice. Scale bars = 100 µm (left) and 25 µm (right) **C** Bar graphs of DHE intensity. All data are presented as mean ± SEM. *P < 0.05, **P < 0.01 compared to the respective control

Oxidative stress and increased ROS played causal roles in the development and maintenance of PTX-induced pain. We investigated whether this was also the case for the antiallodynic effects of KDS2010 on PTX-induced mechanical hypersensitivity (Fig. 4).

First, we investigated whether the analgesic effects of KDS2010 were associated with ROS activity (Fig. 4A). We analyzed the antiallodynic effects of KDS2010 in combination with ROS scavenger, phenyl-N-*t*-butylnitrone (PBN) [29], in PTX and PTX + KDS2010 groups. The antiallodynic effects of PBN (100 mg/kg, i.p) were observed after 0.5 h were decreased at 1 h, and completely disappeared at 6 h, in both the PTX and PTX + KDS2010-treated groups. The peak antiallodynic effects of PBN measured 0.5 h after treatment, did not differ among the groups (Fig. 4A).

Next, we used dihydroethidium (DHE), an oxidative fluorescent dye, to quantify ROS levels in the L4–L6 spinal dorsal horn. PTX-treatment robustly increased the ROS level in spinal dorsal horn of mice, as shown by the increase in DHE-derived fluorescence in the PTX-induced CIPN group compared to the control (Fig. 4B, C, *P* < 0.05). The increased ROS in PTX-treated animals was efficiently attenuated by KDS2010 (25 mg/kg) on day 10 after KDS2010 treatment (on day 21 following PTX treatment in each group). These results suggested that the ROS level was involved in the anti-allodynic effects of the MAO-B inhibitor KDS2010 in PTX-induced CIPN animals.

### KDS2010 restored PTX-induced attenuation of synaptic, but not tonic, GABAA inhibition in SG neurons

ROS involvement in pain is related to diminished inhibitory transmission [10, 16]. Given that KDS2010 showed antiallodynic effects with ROS suppression, we investigated whether KDS2010 restored the attenuated phasic and tonic GABA_A_R currents in the PTX group.

**Fig. 5:**
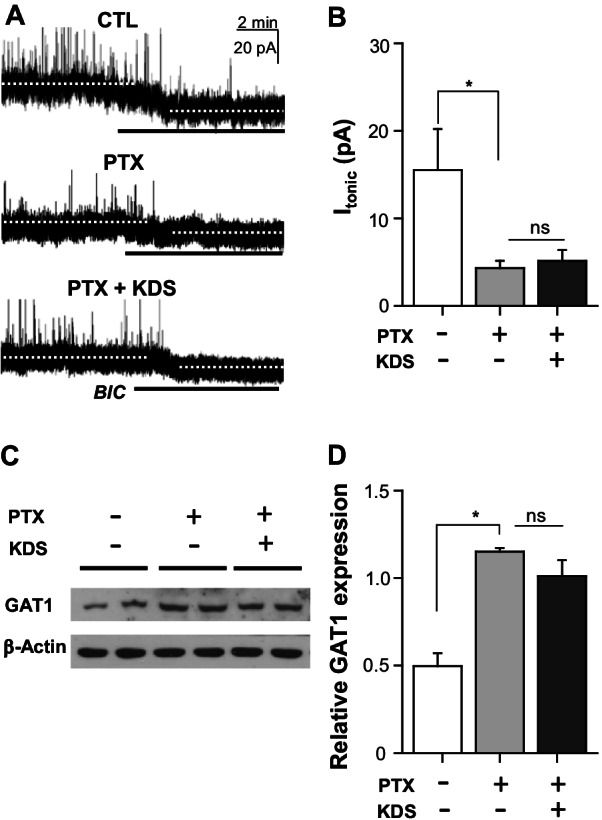
Paclitaxel-induced altered tonic GABA_A_ inhibition and GAT1 expression were not reversed by KDS2010. **A** Representative traces of tonic current in recorded in substantia gelatinosa (SG) neurons in control, PTX-, and KDS2010 (PTX + KDS)-treated mice. Tonic current was significantly reduced by PTX, and KDS2010 failed to reverse this effect. **B** Bar graph summarizing the mean tonic current amplitude. **C** Representative image of Western blot showing GAT-1 expression in the spinal cord of the control, PTX-, and KDS2010 (PTX + KDS)-treated mice. **D** Relative expression of GAT-1, shown by bar graphs. All data are presented as mean ± SEM. *P < 0.05, compared to control group

In addition to GABA_A_ synaptic transmission, PTX reduced GABA_A_ tonic inhibition in the neurons of the spinal dorsal horn. As in a previous report [18], PTX reduced tonic GABA_A_ inhibition, as shown by greater reductions in holding current changes by the GABA_A_R antagonist BIC (20 µM) in the PTX than control group (Fig. 5). Interestingly, KDS2010 failed to affect tonic GABA_A_ inhibition in the PTX group (Fig. 5A, B). Similarly, PTX increased GAT1 expression in the spinal cord, and KDS2010 failed to affect protein expression in the PTX group (Fig. 5C, D).

In agreement with a previous report [30], PTX decreased the presynaptic GABA release, shown by a decrease in GABAergic spontaneous inhibitory postsynaptic current (sIPSC) frequency in the PTX group compared to the control. We analyzed the characteristics of sIPSC, our results showed that sIPSC frequency was significantly restored in the PTX + KDS2010 group (Cont; 0.39 ± 0.10 Hz vs PTX; 0.04 ± 0.01 Hz vs PTX + KDS; 0.3 ± 0.09 Hz, *p* = *0.01,* one-way ANOVA with Tukey *post*-*hoc* test, CTL vs PTX; *p* = *0.02 and PTX vs KDS* + *PTX; p* = *0.03*), although it did not recover to the control level. Neither PTX nor KDS2010 affected the sIPSC amplitude (Cont; 23.54 ± 1.83 pA vs PTX; 19.75 ± 0.92 pA vs PTX + KDS; 21.20 ± 1.04 pA, *p* = *0.14*, one-way ANOVA) and decay time (Cont; 26.62 ± 2.16 ms vs PTX; 28.66 ± 4.88 ms vs PTX + KDS; 25.35 ± 2.28 ms, *p* = *0.75,* one-way ANOVA). These results suggest that the attenuated inhibitory GABA synaptic transmission in the spinal cord of PTX-treated mice was partially recovered by KDS2010.

**Fig. 6:**
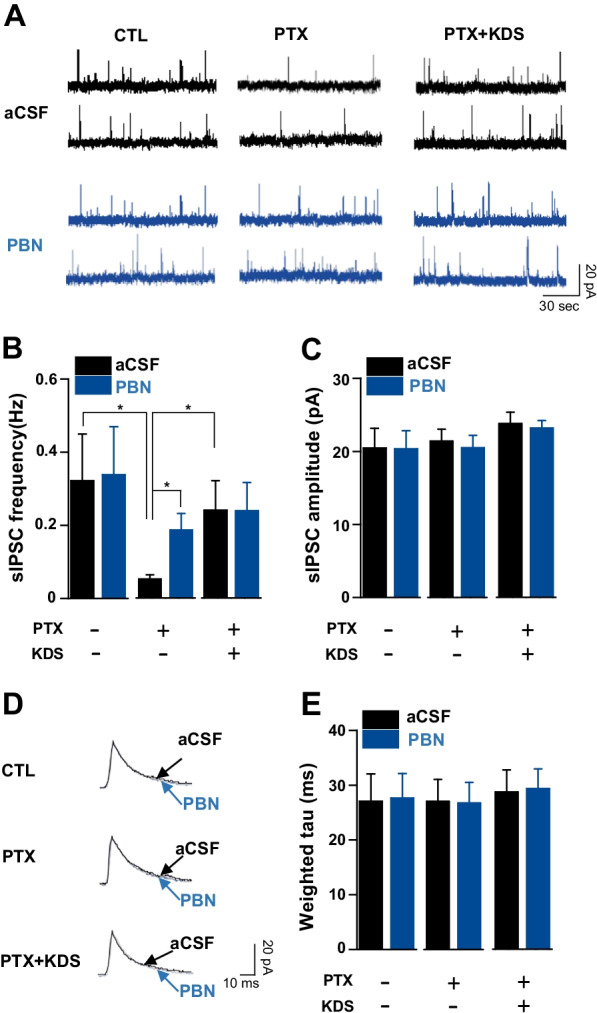
Effects of ROS scavengers on sIPSC in control, PTX and PTX + KDS2010-treated mice. **A** Representative traces of spontaneous inhibitory postsynaptic currents (sIPSCs) before and after application of PBN in control, PTX- and KDS2010-(PTX + KDS) treated mice. **B**, **C** Mean sIPSC frequency and amplitude are summarized in bar graphs, respectively. **D** Averaged sIPSC obtained from same neurons before and after application of PBN in control, PTX- and PTX + KDS2010- treated mice and **E** the mean of decay time of sIPSCs are summarized in bar graphs respectively. All data are presented as mean ± SEM. *P < 0.05 compared to the respective control

To further support our hypothesis that KDS2010 ultimately acts on ROS-GABA synaptic transmission, we investigate the effect of ROS scavenger (PBN) on sIPSC in control, PTX and PTX + KDS2010-treated mice. PBN significantly increased sIPSC frequency in PTX-treated mice while no effect on sIPSC frequency in control and PTX + KDS2010-treated mice (Fig. 6A, B). The sIPSC amplitude and decay time were unaffected by PBN in all tested groups (Fig. 6C–E). Overall, PBN showed comparable effect with KDS2010 on sIPSC frequency, which further suggest that KDS2010 restores the sIPSC frequency by targeting ROS.

### Treatment with KDS2010 had limited side effects on essential brain functions

**Fig. 7:**
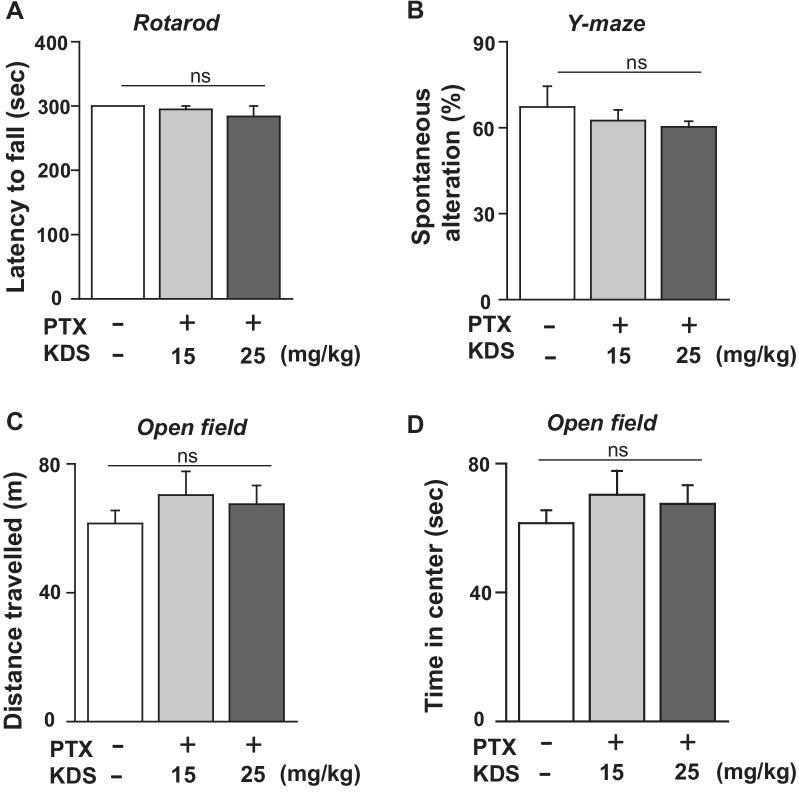
KDS2010 treatment had limited side effects on essential brain functions. **A** The fixed-speed rotarod test was performed in 5-min sessions. Summary bar graphs showing latency to a fall in the controls and mice treated with different concentrations of KDS2010 (PTX + KDS). **B** Working memory was assessed using a Y-maze apparatus. Percentages of spontaneous alterations are shown by bar graphs. **C** Locomotor activity was assessed using an open field test. Summary data showing the total distance travelled and **D** time spent in the center of the maze by each group. All data are presented as mean ± SEM

In addition to body weight loss, analgesic drugs can affect brain functions [31]. We next investigated whether KDS2010, at the dose used to inhibit PTX-induced mechanical hypersensitivity without weight loss, affected essential brain functions (Fig. 7).

KDS2010 (15 and 25 mg/kg) did not significantly affect the latency to fall in the rotarod test (Fig. 7A), suggesting that KDS2010 at these concentrations does not affect motor coordination. In a Y maze test, the same concentrations of KDS2010 did not affect working memory, as shown by the absence of changes in spontaneous alteration (Fig. 7B). In addition, the open field test showed that KDS2010 did not affect locomotor activity or induce anxiety-like behavior (Fig. 7C, D). Overall, these results showed that KDS2010, at 15 and 25 mg/kg, significantly attenuated PTX-induced mechanical hypersensitivity with minimal effect on essential brain functions and body weight.

## Discussion

The present study demonstrated that KDS2010, a newly developed reversible MAO-B inhibitor, reduced PTX-induced mechanical hypersensitivity in a concentration-dependent manner. The antiallodynic effects of KDS2010 were in keeping with the suppression of reactive astrogliosis and ROS levels in the spinal cords of PTX-induced CIPN animals. KDS2010 further reversed the attenuated synaptic GABA_A_R currents in the SG neurons of PTX-induced CIPN animals. The combination of KDS2010 and PBN failed to exert synergistic antiallodynic effects on PTX-induced mechanical hypersensitivity. Overall, our study confirmed that a newly developed and reversible MAO-B inhibitor, KDS2010, may have therapeutic potential for the treatment of chemotherapeutic drug-induced neuropathic pain and that MAO-B is a potential therapeutic target for CIPN.

### Role of ROS-GABAergic inhibition in the antiallodynic effects of KDS2010

Several mediators and neurotransmitters participate in central sensitization. PTX-induced mechanical hypersensitivity is associated with spinal oxidative stress, among other factors maintaining central sensitization, which produces abnormal pain in response to Aβ fiber inputs. ROS is involved in this, as neuropathic pain reduces antioxidant activity in the spinal cord [32], and systemic or intrathecal injections of antioxidants relieve allodynia [7, 33]. Our results support the idea that oxidative stress plays an important role in PTX-induced CIPN, and in the antiallodynic effects of KDS010 on chronic pain. Our in vivo results showing that the antiallodynic effects of the ROS scavengers PBN was not additive with those of KDS2010 in PTX-induced neuropathic models suggested that the antiallodynic effect of KDS2010 is related to anti-ROS properties, or to some other property that suppresses ROS levels in the spinal cord. Our ex vivo results showing that KDS2010 restored the altered ROS level and GABAergic IPSCs further suggests that KDS2010 attenuated PTX-induced mechanical hypersensitivity via the ROS and GABA_A_R inhibitory system. It is noteworthy that PTX increased neuronally-derived ROS at day 7, yet ROS levels in microglia and astrocytes were unaltered [34]. However, it is possible that excessive ROS builds up in glial cells leaks to produce neuronal dysfunction in neuropathic pain [7]. Indeed, an ROS donor, tert-butyl hydroperoxide (t-BOOH), significantly decreased the frequency of IPSCs in SG neurons, and this effect was reversed by PBN. Thus, an intrathecal injection of t-BOOH dose-dependently induces mechanical hyperalgesia [10]. Taken together, these results indicate that increased ROS reduces the inhibitory influence of GABA on SG neurons in neuropathic pain [10, 16], which could be involved in the antiallodynic effects of KDS2010.

It is also noteworthy that decreased GABAergic synaptic transmission due to excessive IL-17 levels in the spinal cord has been linked to PTX-induced CIPN [35]. KDS2010 may restore presynaptic GABA release with the suppression of IL-17 release from reactive astrocytes in PTX-treated mice. However, combined with the fact that IL-17 changes GABA_A_R, as shown by the altered amplitude of spontaneous and evoked IPSCs, this idea is in opposition to our results, which showed that KDS2010 failed to affect the amplitude or decay time of sIPSCs. These results suggest that the antiallodynic effects of KDS2010 are produced by the restoration of GABAergic synaptic transmission, with the ROS decrease reflected in restored IPSC frequency rather than GABA_A_Rs activity in SG neurons. This idea is in line with the suggestion that GABAergic precursor cell transplants reverse PTX-induced mechanical hypersensitivity [36].

### Minimal role of tonic GABA inhibition in the spinal cord antiallodynic effects of KDS2010

PTX reduces GABA_A_ tonic inhibition in spinal dorsal horn neurons showing increased GAT-1 expression ([18] and Fig. 5 in the present study). Thus, the GAT-1 inhibitor prevents the development of hyperalgesia and hypersensitivity, as well as suppressing pre-exiting hypersensitivity in PTX-treated mice [37, 38]. However, our results showing that KDS2010 attenuated mechanical hypersensitivity without restoring the reduced tonic GABA_A_ inhibition or increased GAT-1 expression suggested that the antiallodynic effects of KDS2010 in PTX-induced CIPN mice are not essentially mediated by ambient GABA or tonic GABA inhibition in the spinal cord.

Given that KDS2010 prevented reactive astrogliosis and PWF in PTX-induced CIPN, spinal astrocytes could be involved in the antiallodynic effects of KDS2010, as well as in the pathogenesis of PTX-induced neuropathy. Reactive astrocytes have been reported to release more gliotransmitters [39], raising the possibility that altered GABA release by spinal astrocytes may be involved in some forms of pain and analgesia [40]. The idea that tonic GABA_A_ inhibition is reduced in the spinal cord of PTX-induced CIPN was challenged. Combined with the result showing that KDS2010 did not affect tonic GABA_A_ inhibition in PTX-induced CIPN, these results are in line with the suggestion that compounds selectively upregulating synaptic GABA_A_Rs activity constitute a novel class of analgesics suitable for the treatment of chronic pain [41].

### Astrocytic GABA: role in the antiallodynic effects of KDS2010

Glial GABA synthesized by MAO-B mediates tonic GABA_A_ inhibition, even in the normal cerebellum [42]. The astrocytic GABA release regulating tonic GABA_A_ inhibition is also involved in the pathophysiology of various brain diseases [15, 43–45], and KDS2010 consistently suppresses tonic GABA release from reactive astrocytes in brain diseases [14, 15]. Thus, it is interesting that KDS2010 did not affect tonic GABA inhibition in the spinal cord of PTX-induced CIPN, although it rescued reactive gliosis and pain responses. This apparent discrepancy may be reconciled by the fact that aberrant GABA from reactive astrocytes tonically inhibits the excitability of neighboring neurons in the damaged brain region, whereas tonic GABA_A_ inhibition is under the tight control of GABA transporter activity, clearing GABA from the extracellular space into spinal cord cells in PTX-induced CIPN [18]. Thus, despite its antiallodynic effects, KDS2010 did not affect GAT-1 expression or tonic GABA_A_ inhibition in the spinal cords of PTX-treated mice.

Overall, our results support a dominant role of presynaptic GABA release over astrocytic GABA release in the spinal cord in the antiallodynic effects of KDS2010, in the context of PTX-induced tactile hypersensitivity. However, in addition to the spinal cord, a reduction in GABA levels may contribute to CIPN by decreasing inhibitory signaling in the brain. A decrease in GABA in the thalamus and dorsolateral PAG was accompanied by signs of oxaliplatin-induced CIPN [46, 47]; thus, GABA_A_R activation in PAG reduced CIPN symptoms [47]. Future studies are warranted to investigate the role of astrocytic GABA release in the brain in the antiallodynic effects of KDS2010 on CIPN.

### Minimal side effects of supra-spinal inhibition in the context of the antiallodynic effects of KDS2010

Although increased dopamine in the CNS could mediate the analgesic effects [48], our results showing that KDS2010 did not affect mood, or motor and locomotor activity, argues against the idea that the antiallodynic effects of KDS2010 are a result of increased dopamine in the brain. However, increased brain dopamine may play a role in the antiallodynic effects of KDS2010 in PTX-induced mechanical hypersensitivity, as in other animal models of pain [49]. In the present study, we could not completely exclude the possibility that supra-spinal regulation was indirectly involved in the antiallodynic effects of KDS2010 in PTX-induced CIPN. Further studies are warranted to investigate the antiallodynic effects of KDS2010 in pain models associated with depression, in addition to neuropathic pain models.

In general, long-term MAO inactivation by irreversible inhibitors can be associated with serious side effects or drug–drug interactions; thus, reversible MAO inhibitors have a more favorable side effect profile. Our results showing that KDS2010 reversibly inhibited PTX-induced mechanical hypersensitivity suggest that KDS2010 is a reversible and selective MAO-B inhibitor [14]. The reversibility of weight loss by KDS2010 in the present study may be also due to the reversible effect of KDS2010 on MAO-B. Our results suggest that KDS2010, as a reversible and selective MAO-B inhibitor, is a drug candidate for CIPN and circumvents the shortcomings of irreversible inhibitors, as seen in brain diseases [14].

## Conclusion

Taken together, our results showed that KDS2010, a MAO-B inhibitor, inhibited the development and maintenance of PTX-induced CIPN by restoring altered ROS-GABAergic inhibition in spinal dorsal horn neurons. These results confirmed that the novel MAO-B inhibitor KDS2010 is a potential therapeutic candidate for chemotherapy-induced neuropathic pain, and supported the idea that MAO-B plays a critical role in the development of neuropathic pain. Thus, MAO-B inhibitors have therapeutic potential for chronic pain [12].

## Data Availability

The datasets used and/or analyzed during the current study area available from the corresponding author on reasonable request.

## Abbreviations

ACSF: Artificial cerebrospinal fluid
BIC: Bicuculline
CIPN: Chemotherapy-induced peripheral neuropathy
GABA_A_: γ-Aminobutyric acid type A
GAT1: GABA transpoerter1
GFAP: Glial fibrillary acidic protein
MAO-B: Monoamine Oxidase B
PBN: Phenyl-N-*t*-butylnitrone
PBS: Phosphate buffered saline
PTX: Paclitaxel
PWF: Paw withdrawal frequency
ROS: Reactive oxygen species
SG: Substantia gelatinosa
sIPSC: Spontaneous inhibitory postsynaptic current

## Declarations

### Author information

Su Eun Park, Chiranjivi Neupane and Chan Noh have equally contributed to this work.
